# AI-Driven Multimodal Brain-State Decoding for Personalized Closed-Loop TENS: A Comprehensive Review

**DOI:** 10.3390/brainsci15090903

**Published:** 2025-08-23

**Authors:** Jiahao Du, Shengli Luo, Ping Shi

**Affiliations:** Institute of Rehabilitation Engineering and Technology, University of Shanghai for Science and Technology, Shanghai 200093, China; dujh@usst.edu.cn (J.D.);

**Keywords:** Transcutaneous Electrical Nerve Stimulation (TENS), Brain-State Decoding, Multimodal Neuroimaging, Closed-Loop Neuromodulation, Personalized Neuromodulation

## Abstract

Chronic pain is a dynamic, brain-wide condition that eludes effective management by conventional, static treatment approaches. Transcutaneous Electrical Nerve Stimulation (TENS), traditionally perceived as a simple and generic modality, is on the verge of a significant transformation. Guided by advances in brain-state decoding and adaptive algorithms, TENS can evolve into a precision neuromodulation system tailored to individual needs. By integrating multimodal neuroimaging—including the spatial resolution of functional magnetic resonance imaging (fMRI), the temporal sensitivity of an Electroencephalogram (EEG), and the ecological validity of functional near-infrared spectroscopy (fNIRS)—with real-time machine learning, we envision a paradigm shift from fixed stimulation protocols to personalized, closed-loop modulation. This comprehensive review outlines a translational framework to reengineer TENS from an open-loop device into a responsive, intelligent therapeutic platform. We examine the underlying neurophysiological mechanisms, artificial intelligence (AI)-driven infrastructures, and ethical considerations essential for implementing this vision in clinical practice—not only for chronic pain management but also for broader neuroadaptive healthcare applications.

## 1. Introduction

Despite decades of clinical implementation, pain management remains among the least personalized domains in medicine. Transcutaneous Electrical Nerve Stimulation (TENS), a widely used non-pharmacological intervention, epitomizes this gap: while it is applied ubiquitously, its underlying mechanisms remain unclear, and its clinical outcomes vary significantly across individuals [1]. This disconnect stems from a fundamental mismatch between the static design of conventional TENS protocols and the inherently dynamic, brain-wide nature of chronic pain, thereby constraining its therapeutic potential [2,3].

This limitation is not merely a hardware constraint but a conceptual one. Traditional TENS approaches treat pain as a peripheral input-output problem, focusing solely on the modulation of nociceptive signals. However, pain is not simply a peripheral signal; it is a complex brain state influenced by attention, emotion, and contextual factors [4,5]. This raises critical questions: What if TENS could dynamically adapt to an individual’s brain state in real time [6]? What if stimulation parameters could be aligned precisely with neurophysiological needs as they fluctuate [7]?

In this comprehensive review, we argue that TENS should be re-envisioned not as a generic, one-size-fits-all electrotherapy but as a platform for intelligent, personalized neuromodulation [8,9,10]. We propose that its future lies in the integration of brain-state decoding, real-time feedback, and closed-loop control, enabled by multimodal neuroimaging and advanced computational models. This shift requires a fundamental reframing of not only the technical implementation but also the therapeutic logic underlying TENS.

**Figure 1:**
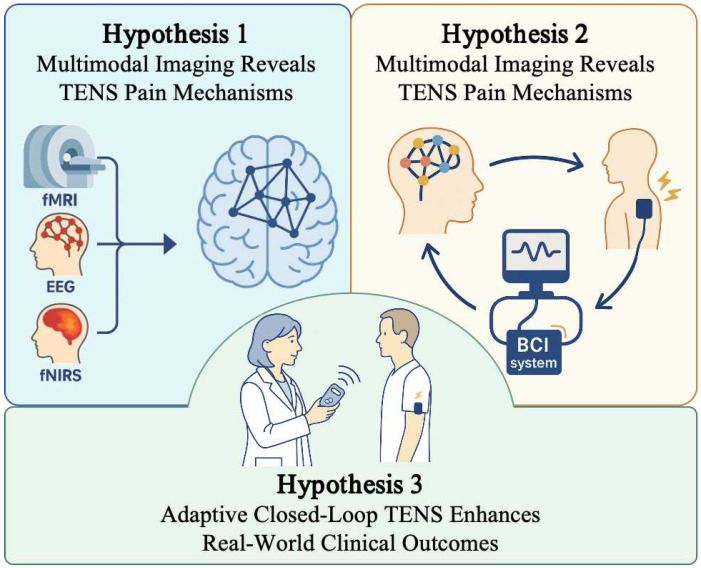
Overview of the three hypotheses supporting adaptive closed-loop TENS for personalized neuromodulation.

We outline three central hypotheses that form the foundation of this vision (Figure 1), integrating multimodal imaging, brain-state decoding, and adaptive closed-loop TENS into a unified framework.

**Hypothesis** **1:** *Multimodal neuroimaging can elucidate unified mechanisms of TENS across spatial, temporal, and ecological dimensions*.

**Hypothesis** **2:** *Brain-state decoding can enable real-time prediction of individual responses to TENS*.

**Hypothesis** **3:** *Adaptive, closed-loop TENS systems will outperform static stimulation protocols in efficacy, specificity, and scalability*.

This review is presented as a comprehensive narrative review. Literature selection was guided by thematic relevance to multimodal brain-state decoding, AI-based inference, and closed-loop TENS, as well as representativeness across neuroimaging and neuromodulation subfields. Instead of a rigid, protocol-driven search strategy, studies were included based on conceptual significance and their contribution to an integrative translational perspective.

We contend that this framework can transform TENS from a generic, open-loop device into a brain-aware, precision-directed neuromodulation system, with implications extending beyond chronic pain management to broader neuroadaptive healthcare applications.

## 2. Hypothesis 1: Multimodal Imaging Can Reveal Unified Mechanisms of TENS-Induced Pain Modulation

Pain is not merely a sensory input; it is a distributed, fluctuating brain state shaped by attention, emotion, memory, and context [11,12]. While TENS interfaces with this system peripherally, its brain-level effects remain inconsistently characterized across studies and modalities. To move beyond fragmented insights, we hypothesize that integrating multimodal imaging—combining the spatial specificity of fMRI [13], the temporal sensitivity of the EEG [14], and the ecological validity of fNIRS [15,16]—can reveal convergent mechanisms underlying TENS-induced analgesia. This systems-level approach could shift TENS from empirical application toward mechanism-informed personalization.

### 2.1. Mechanistic Insights Across Scales and Contexts

Functional MRI studies consistently demonstrate that TENS modulates large-scale brain networks involved in pain regulation [17,18]. Specifically, it enhances top-down control through strengthened connectivity between the anterior cingulate cortex and prefrontal regions while concurrently reducing hyperactivity in the insula and default mode network—patterns associated with chronic pain persistence [19]. Graph-theoretical analyses further indicate that TENS increases network efficiency and modularity within salience and limbic networks, aligning with clinical symptom relief [20].

Complementary insights from the EEG capture cortical dynamics at millisecond resolution. Increases in frontal alpha power reflect enhanced cortical inhibition, while elevated gamma coherence across somatosensory and prefrontal areas suggests improved attentional regulation [21,22]. Simultaneous EEG-fMRI studies reveal that these oscillatory changes often precede downstream thalamic suppression, supporting a hierarchical model of neuromodulation in which cortical entrainment initiates subcortical recalibration [23].

Meanwhile, fNIRS extends this mechanistic perspective into naturalistic contexts. During real-world tasks and clinical interactions, TENS consistently elevates prefrontal oxygenation—a biomarker linked to analgesia and reduced anxiety [24]. Additionally, dyadic fNIRS recordings have shown increased clinician–patient neural synchrony during TENS sessions, indicating that interpersonal and contextual factors shape the brain’s responsiveness to neuromodulation [25].

Collectively, these modalities provide complementary perspectives, mapping structural reconfiguration, dynamic oscillatory modulation, and social-contextual entrainment during TENS. Their convergence supports a unified neurophysiological framework in which TENS modulates pain not at a single node but across a distributed, adaptive brain network.

Multimodal imaging thus serves not merely as a measurement tool but as a scaffold for decoding TENS-induced brain changes across functional layers. By aligning spatial precision, temporal resolution, and contextual relevance, this approach can transform TENS from a black-box technique into a theory-guided, precision-targeted intervention.

### 2.2. Toward a Unified Framework of Pain Modulation

While individual imaging modalities each provide valuable insights into TENS-induced brain activity, their true potential lies in integration. A unified framework should explain not only where TENS acts but also when, for whom, and under what contextual conditions.

Multimodal data increasingly support a two-stage model of TENS-induced neuromodulation [26,27]. First, rapid cortical entrainment—indexed by frontal alpha suppression and gamma synchronization—primes top-down regulatory pathways. This is followed by downstream modulation of deeper affective and sensory hubs, including the insula and thalamus, as demonstrated in fMRI connectivity studies [28]. Such cascades align with dynamic, hierarchical models [29] of pain processing, wherein superficial cortical patterns scaffold deeper network recalibration.

Crucially, cross-modal biomarkers of effective modulation are emerging. For instance, gamma coherence measured via the EEG correlates with prefrontal hemodynamic activation captured by fNIRS, while decreased insular activity observed in fMRI mirrors frontal desynchronization in the EEG [30,31,32,33]. These cross-validated markers may serve as robust signatures of therapeutic engagement, forming candidates for real-time monitoring and personalization algorithms.

Dynamic functional connectivity (DFC) offers another lens to assess TENS efficacy. Pain-related networks reconfigure over seconds in response to internal states and external interventions. TENS appears to enhance network flexibility and switching capacity within the default mode and salience networks, features linked to improved clinical outcomes [34,35]. This suggests that neuromodulatory responsiveness—not just static baseline structures—may underlie successful treatment outcomes.

Real-time analysis of EEG and fNIRS signals is also enabling the development of closed-loop systems, wherein stimulation adapts to moment-to-moment brain activity [36,37,38]. While technically complex, these advances are pushing TENS toward an intelligent architecture. However, this evolution raises ethical and operational challenges: Who controls the feedback loop [39,40]? What defines a meaningful neural threshold for adaptation? Transparency, safety, and explainability must be integral to any system that modulates the brain based on its own activity.

A unified model of TENS neuromodulation is thus emerging—one that integrates timing, topology, and plasticity. The brain’s responsiveness to stimulation is not fixed but state-dependent and adaptive [41]. To harness this, we must advance from using imaging solely for observation toward leveraging imaging for control.

## 3. Hypothesis 2: Brain-State Decoding Can Enable Real-Time Prediction of Individual Responses to TENS

The inconsistent efficacy of TENS across individuals has long undermined its clinical credibility. However, this variability may not represent a flaw in the technology itself but rather reflect unmeasured brain-state heterogeneity. The promise of precision neuromodulation lies in recognizing this heterogeneity and adapting stimulation protocols in real time. Brain-state decoding, enabled by advanced machine learning methods, offers the tools necessary to identify individual neural signatures and to build predictive, responsive stimulation systems, shifting TENS from passive delivery toward intelligent personalization.

### 3.1. Brain-State Decoding for Predicting TENS Responsiveness

Deep neural networks have significantly advanced our capacity to extract latent features from high-dimensional neural data. Convolutional architectures applied to fMRI and fNIRS can identify spatial activation patterns in regions such as the prefrontal cortex and insula, while recurrent models capture the dynamic aspects of EEG signals, including gamma synchrony and alpha suppression [15,42,43]. These neural signatures correlate with therapeutic outcomes and hold promise as biomarkers of TENS responsiveness.

Beyond feature extraction, predictive modeling enables patient stratification and individualized intervention planning. Supervised learning methods, including support vector machines and ensemble classifiers, can classify likely responders versus non-responders, informing pre-treatment decisions. Meanwhile, generative approaches, such as variational autoencoders and generative adversarial networks, can augment small datasets and synthesize plausible brain-state variants to enhance model generalizability. This represents a conceptual shift from treating trial-by-trial variability as noise to recognizing meaningful structure within individual differences.

The success of TENS may hinge less on the specific stimulation parameters and more on neural readiness for modulation. Machine learning reframes the therapeutic question from “what to stimulate” to “when and in whom to stimulate.”

### 3.2. Adaptive Closed-Loop Control Strategies

Given that brain states fluctuate from moment to moment, stimulation strategies should adapt correspondingly. Real-time EEG and fNIRS signals now enable adaptive protocols in which decreases in frontal alpha power or increases in prefrontal oxygenation dynamically trigger adjustments in stimulation amplitude, pulse frequency, or timing. Early studies indicate that such closed-loop systems can outperform static protocols, providing faster and more sustained analgesic effects [44,45].

Reinforcement learning offers a robust framework for optimizing control in uncertain, feedback-rich clinical environments [46,47,48]. Actor–critic models and deep Q networks can iteratively refine TENS delivery by learning which brain-state conditions predict beneficial outcomes. Importantly, these systems do not require extensive labeled datasets upfront, making them well-suited for continuous, personalized adaptation within clinical settings.

By employing closed-loop control, TENS evolves from being merely a stimulator to functioning as a learner, with its operational rules shaped dynamically by the brain’s responses.

### 3.3. Integration with Brain–Computer Interfaces for Precision Neuromodulation

The integration of brain–computer interfaces (BCIs) with machine learning opens new avenues for fine-grained personalization of TENS. Oscillatory signals, such as gamma bursts and theta asymmetries, can inform the timing and targeting of stimulation windows with millisecond precision, modulated by cognitive and emotional states. This level of precision moves TENS beyond generalized therapy toward neuroadaptive co-regulation.

As these systems become increasingly autonomous, ensuring explainability is essential. Both clinicians and patients must understand the rationale behind system actions to foster trust and safe implementation. Techniques such as SHAP (SHapley Additive exPlanations) and layer-wise relevance propagation can enhance model transparency, while hybrid systems that integrate domain knowledge with data-driven models can further improve interpretability without compromising performance [49,50,51,52,53].

When machines are tasked with interpreting and responding to brain states, the challenge extends beyond achieving predictive accuracy to ensuring accountability. Transparent and interpretable models form the foundation for clinical trust in adaptive neurotechnologies.

### 3.4. Methodological Framework for Multimodal Brain-State Decoding and Closed-Loop TENS

**Figure 2:**
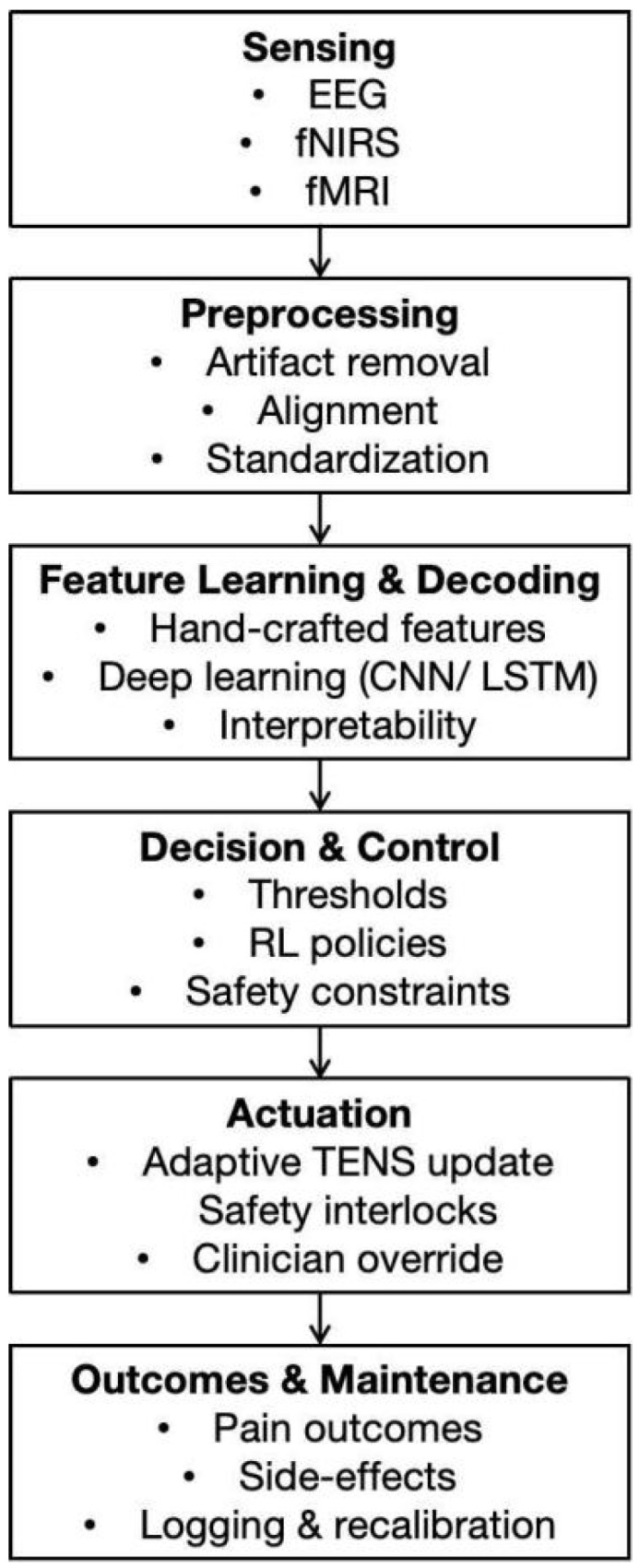
Unified methodological pipeline for multimodal brain-state decoding and adaptive closed-loop TENS neuromodulation.

To synthesize the cross-disciplinary approaches reviewed above, we outline a unified methodological framework (Figure 2) that captures the essential stages required for adaptive closed-loop neuromodulation. The pipeline integrates multimodal sensing, signal conditioning, feature extraction and decoding, adaptive decision policies, and feedback execution, supported by continuous monitoring and model maintenance.

At the sensing layer, neural and physiological signals such as EEG, fNIRS, and fMRI provide complementary perspectives on pain-related brain states, with the EEG and fNIRS further distinguished by their potential for ambulatory and wearable deployment [13,14,15,16,36,37,38,54,55]. Preprocessing and quality control are indispensable to ensure robustness across devices and environments, encompassing artifact suppression, temporal alignment, and channel-level diagnostics [54,55].

On this basis, feature learning strategies span from conventional time–frequency and connectivity metrics [15,42,43] to deep representation learning (e.g., CNN- or LSTM-based architectures) [49,50,51], which enhance state estimation and responder stratification. Transparency techniques, including attribution maps and post hoc model interpretation, support clinical interpretability [52,53].

Decision and control policies translate decoded states into TENS parameter adaptations through thresholding or reinforcement-learning paradigms [46,47,48], while embedding explicit safety constraints and clinician-defined limits [39,40]. The execution layer delivers real-time actuation under safety interlocks, often supported by edge computation for latency-sensitive contexts [54,55]. The framework also integrates longitudinal logging of outcomes, side effects, and signal quality indices to enable continuous recalibration, facilitate reproducibility across sites, and prepare for regulatory evaluation [56,57,58].

## 4. Hypothesis 3: Adaptive Closed-Loop TENS Systems Can Enhance Clinical Efficacy in Real-World Settings

Most neuromodulation therapies currently rely on static stimulation protocols characterized by fixed parameters and delivery schedules, operating under the assumption that the therapeutic target remains constant across time and individuals. However, the brain is inherently dynamic, particularly under chronic pain conditions, where fluctuations in attention, emotion, and network states can modulate responsiveness to neuromodulatory interventions. We hypothesize that adaptive, closed-loop TENS systems, informed by real-time brain and physiological signals, will outperform static stimulation protocols in efficacy, specificity, and scalability by aligning intervention delivery with the dynamic neurophysiological state of each patient.

### 4.1. Architectures for Adaptive, Closed-Loop Neuromodulation

An effective adaptive, closed-loop TENS system requires three essential capabilities: real-time sensing of neural and physiological signals, accurate interpretation of these signals to infer brain state, and dynamic adjustment of stimulation parameters based on the interpreted state. Potential control signals include fNIRS-derived prefrontal activation, EEG alpha desynchronization, gamma synchrony, and connectivity metrics reflecting network reorganization associated with analgesia.

Advances in wearable sensor technology and edge computing now enable these capabilities to be deployed beyond laboratory environments [54,55]. Embedded processing supports low-latency adaptation while maintaining data privacy, and wireless sensor integration allows continuous monitoring during daily activities, facilitating ambulatory neuromodulation essential for chronic pain management.

From an algorithmic perspective, reinforcement learning (RL) offers a powerful framework for continuous, patient-specific adaptation in feedback-rich, data-sparse environments. Actor–critic models and deep Q networks can iteratively refine stimulation strategies based on real-time outcomes, learning personalized intervention patterns rather than relying on static rules. Additionally, unsupervised methods such as clustering can segment patients into neuro-responsive subtypes, enabling population-level presets while allowing fine-tuning within individuals—a dual-layer adaptation critical for scalable precision neuromodulation.

### 4.2. Evaluating the Superiority of Adaptive Closed-Loop TENS over Static Protocols

Adaptive, closed-loop TENS systems demonstrate clear advantages over static protocols. In terms of efficacy, aligning stimulation with optimal neurophysiological states enhances analgesic effects while minimizing unnecessary or mistimed stimulation that can lead to discomfort or neural adaptation. Specificity is improved through real-time interpretation of multimodal neural and physiological signals, enabling targeted delivery when the nervous system is most receptive to modulation.

Scalability is supported by wearable, autonomous systems capable of delivering therapy outside clinical environments, reducing the dependency on clinic-based treatments while maintaining high-quality treatment. Early studies indicate that adaptive protocols, guided by markers such as reduced frontal alpha power or increased prefrontal oxygenation, yield faster and more sustained pain relief compared to static stimulation approaches, underscoring the potential of adaptive systems to improve treatment consistency and responsiveness.

### 4.3. Implementation Pathways: Devices, Interfaces, and Clinical Integration

To transition adaptive, closed-loop TENS systems from experimental prototypes to clinically viable solutions, hardware must be lightweight, wearable, and capable of real-time adaptation while ensuring safety and reliability. Recent prototypes integrate wireless EEG and fNIRS sensors with mobile-connected TENS controllers, utilizing local computation to reduce latency and enhance privacy.

User interface design is critical for clinical acceptance and usability [59,60]. Clinicians require clear, interpretable visualizations of system decisions and the capacity to supervise and override automated actions when necessary. Patients benefit from feedback mechanisms that enhance confidence and engagement without introducing unnecessary complexity. The integration of explainable AI tools, such as SHAP visualizations and signal attribution maps, can bridge the interpretability gap between system actions and clinical oversight, fostering trust in adaptive neuromodulatory interventions.

Validation frameworks must evolve to appropriately assess these systems. Traditional metrics, such as mean pain reduction across trials, may not sufficiently capture adaptability and responsiveness. Evaluation criteria should include temporal stability, responsiveness to patient-specific fluctuations, and the prevention of overfitting to transient states. Algorithms must therefore be evaluated not only on their immediate outcomes but also on how they adapt and maintain performance over time.

### 4.4. From Experimental Systems to Routine Clinical Care

Technological readiness alone does not guarantee clinical adoption. Challenges such as variability in sensor placement, signal noise, and patient movement remain barriers to robustness in real-world applications. Establishing standardized preprocessing pipelines, quality control protocols, and harmonized reference datasets will be critical for reproducibility across sites and patient populations [61,62].

Workflow integration is equally essential. Adaptive TENS systems should seamlessly integrate into existing clinical care pathways without necessitating significant workflow modifications. Automated setup, intuitive interfaces, and minimal training requirements for clinicians and patients are essential to achieving usability without compromising functionality. Early pilot studies demonstrate that co-design approaches involving clinicians and patients can effectively align adaptive TENS systems with practical needs, balancing technical sophistication with ease of use.

Ultimately, the critical question is not whether adaptive, closed-loop TENS systems can function, but whether they can do so seamlessly and reliably in real-world environments. Only through such seamless integration can the enhanced efficacy, specificity, and scalability of these systems translate into meaningful improvements in personalized pain management.

## 5. Comparative Summary of Key Studies

Building on the preceding review of individual modalities, a comparative summary of representative studies is presented below to highlight methodological diversity, analytical strategies, and translational relevance. A comparative synthesis of representative studies on multimodal brain-state decoding and closed-loop TENS is presented in Table 1. The purpose of this comparison is to illustrate the methodological diversity, neural modalities, analytical strategies, and translational stages reflected in the literature.

Several important trends can be observed. First, EEG-based approaches remain predominant, given their portability, high temporal resolution, and relatively low cost. Studies employing EEGs have applied both classical machine learning algorithms, such as support vector machines (SVMs) and random forest, as well as deep learning models, including convolutional neural networks (CNNs) and long short-term memory (LSTM) architectures. Results consistently suggest that advanced deep learning methods achieve higher decoding accuracy, in some cases exceeding 85–90% [18,36].

Second, fMRI-based research contributes mechanistic insights by mapping pain-related networks and identifying predictive biomarkers [23,46]. Despite these strengths, the high financial and logistical requirements restrict their use to research environments rather than routine clinical practice.

Third, fNIRS and hybrid modalities (e.g., EEG–fNIRS integration) demonstrate potential in improving decoding robustness and performance [27,31,58]. These approaches provide non-invasive and comparatively portable solutions, although their reproducibility in real-world scenarios remains to be fully validated.

Finally, clinical pilot investigations exploring closed-loop TENS guided by brain-state decoding are limited in number [60]. Preliminary findings indicate potential benefits for pain alleviation, yet these studies often suffer from small sample sizes, methodological heterogeneity, and inconsistent outcome measures.

Overall, the comparative analysis highlights clear methodological advances but also underscores the lack of standardization across modalities and protocols. The field is still at an early stage of clinical translation, and greater harmonization of experimental designs, performance metrics, and clinical endpoints will be essential for moving toward robust and scalable clinical applications.

## 6. Discussion and Future Outlook

TENS has persisted in clinical practice not because it is deeply understood, but because it is easy to use and difficult to replace. Its analgesic effects—real yet inconsistent—have long resisted mechanistic explanation and predictive control. Yet in this ambiguity lies opportunity: TENS can serve as a scalable testbed for rethinking personalized neuromodulation in the age of neuroimaging and adaptive intelligence.

This review has proposed a translational framework grounded in three interlinked propositions. First, pain is not merely a peripheral signal but a dynamic brain-state phenomenon that must be observed in real time across distributed networks. Multimodal imaging—integrating the spatial precision of fMRI, the temporal acuity of the EEG, and the ecological realism of fNIRS—can reveal convergent, system-level mechanisms underlying TENS-induced analgesia. These insights recast TENS from a black-box technique into a tool for decoding the distributed and context-sensitive nature of pain.

Second, machine learning enables therapy to evolve from a static intervention to a dynamic, state-driven dialogue. Deep neural networks can extract latent features from high-dimensional neural signals, while supervised and generative models enable stratification of responders and augmentation of sparse datasets. More critically, reinforcement learning frameworks can operationalize closed-loop control, allowing TENS systems to adapt stimulation parameters based on moment-to-moment neural states. This transforms the question from “what to stimulate” to “when and in whom,” reframing TENS delivery as a continuously learning, personalized process.

Third, adaptive, closed-loop TENS systems can outperform static protocols in efficacy, specificity, and scalability. By aligning stimulation with the optimal neural states, these systems enhance analgesic outcomes while minimizing unnecessary stimulation, discomfort, and habituation. The integration of wearable, multimodal sensing and edge computing enables real-time monitoring and adaptation in ambulatory environments, bridging the gap between laboratory efficacy and real-world effectiveness. These systems can not only provide therapy but also capture data that refines future interventions, closing the feedback loop between device, brain, and behavior.

Early clinical pilot studies have begun to translate these adaptive paradigms into patient care. For example, EEG-informed closed-loop TENS protocols have been tested in small cohorts of chronic pain patients, showing enhanced analgesic responses compared to open-loop stimulation [60,63]. These investigations demonstrate the feasibility of dynamically aligning stimulation parameters with neural markers of pain reactivity while also revealing challenges such as heterogeneous endpoints, limited sample sizes, and short follow-up durations. Although preliminary, such trials provide proof-of-concept evidence that brain-state-guided closed-loop TENS can be both technically implementable and clinically meaningful, paving the way for larger randomized studies to evaluate generalizability, safety, and long-term impact.

However, several challenges remain. Neuroimaging modalities, while advancing in portability, often remain siloed in research settings, and there is a need to harmonize multimodal data streams for seamless integration into adaptive systems [56,57,58]. Machine learning models, despite their promise, face limitations in generalizability across populations, devices, and clinical contexts, necessitating the development of lightweight, interpretable algorithms suitable for real-time deployment. Regulatory pathways for algorithm-driven, adaptive therapies are nascent, and validation frameworks must evolve beyond traditional outcome metrics to incorporate dynamic measures of responsiveness, stability, and patient-device synchrony. An additional practical challenge lies in energy consumption for closed-loop TENS systems [63]. Unlike static stimulation, adaptive protocols demand continuous sensing, signal processing, and algorithmic decision-making, which collectively increase power requirements. For wearable deployment, battery efficiency and low-power hardware design become critical determinants of usability. Promising strategies include sparse -sampling of neuroimaging inputs, lightweight machine learning models, and hardware-level optimization, all of which aim to balance adaptability with long-term portability in real-world environments. Another instructive reference point comes from closed-loop spinal cord stimulation. Like TENS, closed-loop SCS leverages feedback-driven adaptation to improve efficacy [64]; however, it typically relies on invasive sensing (e.g., evoked compound action potentials) and implantable power sources. In contrast, closed-loop TENS depends on fully non-invasive neural recordings and wearable batteries. This difference highlights complementary roles: SCS offers higher spatial specificity and stability, whereas TENS emphasizes accessibility and scalability. Positioning TENS within this broader neuromodulation landscape underscores its potential not as a replacement but as a parallel pathway toward personalized, adaptive pain management.

These challenges are not purely technical; they are epistemological. We must redefine what counts as “evidence” in adaptive neuromodulation, prioritizing time-resolved responses and network flexibility over static averages. Equally, we must embed explainability, patient autonomy, and informed consent into system design rather than retrofitting them post hoc. As adaptive systems become more autonomous, transparency and accountability will be critical for clinician and patient trust.

While some reviews rely on pooled counts, numerical aggregation, or graphical meta-summaries, such approaches are less applicable to a field as heterogeneous as multimodal neuroimaging and adaptive TENS. The studies surveyed here differ widely in methodology, patient population, data modality, and analytic pipeline, making simple quantitative synthesis potentially misleading. Instead, this review adopts a qualitative and integrative strategy. The comparative table distills methodological breadth and cross-modal diversity, while the narrative synthesis identifies recurring conceptual patterns and translational implications. This structured but non-numerical approach provides clarity without overstating precision, ensuring that readers can appreciate both the diversity of methods and the coherence of emerging themes.

The future of TENS as a platform for adaptive neuromodulation research will depend on interdisciplinary collaboration among engineers, clinicians, and patients. Workflow integration must prioritize usability, requiring interfaces that are intuitive and setups that demand minimal disruption to existing clinical practices. Co-design approaches can ensure that technological sophistication aligns with practical needs, enhancing acceptance and scalability.

As TENS enters the era of adaptive intelligence, its value may lie less in its electrodes and parameters and more in its potential to illuminate how the brain, machine, and behavior interact in real time. It offers a rare opportunity to study neuromodulation as a dynamic dialogue rather than a one-way intervention. Looking forward, future research should prioritize multicenter randomized controlled trials to validate these concepts, the establishment of standardized multimodal datasets, the development of lightweight and interpretable real-time algorithms, and the integration of patient-centered co-design strategies to facilitate clinical translation. By embracing this potential, TENS can transform from a widely used but poorly understood tool into a model system for precision, adaptive, and personalized neuromodulation, advancing the science and practice of pain management in the process.

## 7. Conclusions

TENS stands at the threshold of evolving from a static, empirical modality into an adaptive, precision-guided neuromodulation system. By integrating multimodal neuroimaging, machine learning, and wearable sensing technologies, TENS can match the complexity of pain with intelligent, state-responsive interventions that adjust in real time to individual brain states. This review has outlined a translational pathway toward this future, emphasizing how multimodal decoding, adaptive closed-loop control, and reinforcement learning can transform TENS into a scalable model for personalized pain management while advancing our mechanistic understanding of neuromodulation.

Yet this shift will not occur automatically. It demands conceptual clarity, technical integration, and ethical foresight, ensuring that adaptive systems remain interpretable, safe, and patient-centered. As TENS enters the era of adaptive intelligence, its greatest value may lie not only in providing more consistent analgesia but in serving as a testbed for reimagining neuromodulation as a real-time dialogue between the brain, machine, and behavior. Precision does not require complexity. Sometimes, it only requires listening better—and responding faster.

## Figures and Tables

**Table 1:** Representative studies on multimodal brain-state decoding and closed-loop TENS, categorized by modality, analytical method, dataset characteristics, and key performance indicators.

Modality	Representative References	Analytical Method	Data Characteristics	Key Findings/Performance
EEG	[18,36,41]	SVM, RF, CNN, LSTM	20–60 subjects, pain task paradigms	CNN/LSTM achieve >85% accuracy in pain state classification; classical ML ~70–80%
fMRI	[23,46,51]	Multivariate pattern analysis, regression	Small cohorts (*n*=12–30), high spatial resolution imaging	Identification of pain-related networks and predictive biomarkers; limited by cost and accessibility
fNIRS	[27,31,58]	Logistic regression, hybrid ML models	10–25 subjects, cortical hemodynamic responses	Effective in differentiating pain vs. no-pain states; moderate accuracy (~75–85%)
EEG–fNIRS (Hybrid)	[31,58]	Multimodal fusion, ensemble learning	Dual-modality datasets	Improved robustness and performance compared with unimodal approaches
Clinical closed-loop TENS pilot	[60,63]	Adaptive algorithms, feedback-based control	Pilot cohorts (n < 20)	Initial evidence of analgesic benefit; limited by heterogeneity and small sample sizes

## Data Availability

No new data were created or analyzed in this study.
